# Proteins of *Leishmania (Viannia) shawi *confer protection associated with Th1 immune response and memory generation

**DOI:** 10.1186/1756-3305-5-64

**Published:** 2012-03-30

**Authors:** Luiz Felipe D Passero, Ana Kely Carvalho, Maria LAC Bordon, Alexis Bonfim-Melo, Karina Carvalho, Esper G Kallás, Bianca BA Santos, Marcos H Toyama, Adriana Paes-Leme, Carlos EP Corbett, Márcia D Laurenti

**Affiliations:** 1 Depto. de Patologia da Faculdade de Medicina da Universidade de São Paulo, Laboratório de Patologia de Moléstias Infecciosas (LIM-50), São Paulo, Brazil; 2 Campus Experimental do Litoral Paulista, Universidade Estadual Paulista, São Vicente, São Paulo, Brazil; 3 Division of Clinical Immunology and Allergy, (LIM-60), University of São Paulo, São Paulo, Brazil; 4 Brazilian Biosciences National Laboratory, CNPEM, Campinas, Brazil; 5 Depto. de Patologia da Faculdade de Medicina da Universidade de São Paulo, Laboratório de Patologia de Moléstias Infecciosas (LIM-50), Av. Dr. Arnaldo, 455, Cerqueira César, SP 01246-903, Brazil

**Keywords:** *Leishmania (Viannia) shawi*, Proteic fraction, Immunization, Cellular immune response, Long-term protection

## Abstract

**Background:**

*Leishmania (Viannia) shawi *parasite was first characterized in 1989. Recently the protective effects of soluble leishmanial antigen (SLA) from *L. (V.) shawi *promastigotes were demonstrated using BALB/c mice, the susceptibility model for this parasite. In order to identify protective fractions, SLA was fractionated by reverse phase HPLC and five antigenic fractions were obtained.

**Methods:**

F1 fraction was purified from L. (V.) shawi parasite extract by reverse phase HPLC. BALB/c mice were immunized once a week for two consecutive weeks by subcutaneous routes in the rump, using 25 μg of F1. After 1 and 16 weeks of last immunization, groups were challenged in the footpad with L. (V.) shawi promastigotes. After 2 months, those same mice were sacrificed and parasite burden, cellular and humoral immune responses were evaluated.

**Results:**

The F1 fraction induced a high degree of protection associated with an increase in IFN-γ, a decrease in IL-4, increased cell proliferation and activation of CD8^+^T lymphocytes. Long-term protection was acquired in F1-immunized mice, associated with increased CD4^+ ^central memory T lymphocytes and activation of both CD4^+ ^and CD8^+ ^T cells. In addition, F1-immunized groups showed an increase in IgG2a levels.

**Conclusions:**

The inductor capability of antigens to generate memory lymphocytes that can proliferate and secrete beneficial cytokines upon infection could be an important factor in the development of vaccine candidates against American Tegumentary Leishmaniasis.

## Background

Leishmaniasis constitutes a group of diseases ranging from visceral to cutaneous forms of illness. In the New World, different species of *Leishmania *act as agents of human disease [1,2], such as *L. (L.) chagasi *or *L. (L.) infantum*, which is the only species known to induce the visceral form of the disease. Tegumentary forms can be caused by distinct species, which are responsible for the spectrum of disease ranging from single cutaneous lesions to anergic diffuse leishmaniasis [3-5]. The control of tegumentary leishmaniasis in the New World is difficult due to the natural features of reservoir and vectors, making the elimination of both components hard to achieve [6]. For these reasons, the development of prophylactic measures is highly indicated for the control of leishmaniasis.

An interesting prophylactic measure to limit the epidemiology of leishmaniasis is the development of vaccines. The immunogens used to formulate vaccine candidates can be classified according to their method of formulation: live parasites, the classic model of leishmanization [7]; first generation vaccines that use crude parasite antigens [8,9]; second generation vaccines that use fractionated, purified or recombinant antigens [10]; and third generation vaccines that use genetic material as the immunogen [11].

Second generation vaccine candidates present good perspectives for the development of vaccines, since some immunosuppressive antigens present in first generation vaccines can be eliminated through purification [12]. Moreover, second generation vaccines present no risk of intercalating with the host genetic material, as some DNA vaccines can do, despite their potential for curing a number of disorders [13]. Thus, an important class of second generation vaccine candidates have been purified and analyzed regarding their protective properties, such as fucose mannose ligand and antigens released by visceral and cutaneous strains of *Leishmania sp*., which induced strong protection in experimental and natural leishmaniasis [14-16].

In the New World, at least seven species of *Leishmania *affect humans and the most important cutaneous species are *L. (L.) amazonensis *and *L. (V.) braziliensis *[1]. For this reason, the development of vaccine candidates is important to protect people living in endemic areas who are exposed to vectors and parasites [17]. A series of fractions and purified antigens have been characterized and used to achieve protection against *L. (L.) amazonensis *and *L. (V.) braziliensis *[18-20]. Despite their medical and epidemiological importance in the New World, other parasite species that affect humans are rarely studied, such as *L. (V.) shawi *and *L. (V.) panamensis *[21,22]. Some recent studies have demonstrated that antigens derived from both these species were immunogenic and beneficial to experimental hosts following challenge [23,24]. Species of the *Viannia *subgenus can be a useful target for developing cross-protective vaccine candidates, since they are monophyletic, and thus have homologous antigens with other *Leishmania *(*Viannia*) sp. [25], facilitating the development of cross-protective vaccines. Moreover, in the New World, the majority of species affecting humans belong to the *Viannia *subgenus, thus justifying the search of vaccine candidates among *L. (Viannia*) sp. representatives.

In order to identify immunogenic fractions involved in the protection of BALB/c mice, the soluble leishmanial antigen (SLA) from *L. (V.) shawi *was fractionated and the effect of one proteic fraction (F1) was analyzed regarding its constitution and the degree of protection induced in BALB/c mice following an infectious challenge. The main immunological alterations that occurred in BALB/c mice were also evaluated.

## Methods

### Experimental animals

Eight-week-old male BALB⁄c mice obtained from the Animal Facility of the School of Medicine of São Paulo University, Brazil, were maintained in our laboratory during the experiments, in accordance with the institutional guidelines regarding the welfare of experimental animals and with the approval of the Animal Ethics Committee of São Paulo University (0280/07).

### Parasite

*L. (V.) shawi *(MHOM/BR/96/M15789) parasite was isolated from a patient with American tegumentary leishmaniasis in Buriticupu County, State of Maranhão, Brazil, and identified by monoclonal antibodies and multilocus enzyme electrophoresis at the Evandro Chagas Institute in Belém, State of Pará, Brazil. The parasites maintained in BALB/c mice footpads were isolated and grown in RPMI-1640 medium (Gibco Invitrogen, USA) supplemented with 10% heat-inactivated FCS, 0.25 mM HEPES, 10 μg/ml gentamicin and 100 IU/ml penicillin. On day 6 of culture, promastigote forms were centrifuged (1,200 *g*, 10 min) with phosphate buffer saline solution (PBS, pH 7.4) and used for antigen production and mouse infection.

### Purification of antigens from *L. (V.) shawi *promastigotes

Promastigote forms (~10^9 ^promastigotes) in the stationary phase of growth were recovered by centrifugation at 1,200 *g *for 10 min at 4°C, followed by 3 washes with phosphate buffered saline (PBS) at 1,200 *g *for 10 min at 4°C. Lysis buffer (20 mM Tris-HCl; 40 mM NaCl; 10 mM EDTA) was added to the promastigote pellet and the material was frozen in liquid nitrogen and thawed at room temperature three times to produce total parasite extract (AG). Further, AG was centrifuged at 10,000 *g *for 1 h at 4°C, the supernatant was collected (SLA), lyophilized and applied onto a reverse phase HPLC to purify the proteic fractions, according to the methodology described in Toyama *et al. *(2001) [26]. Briefly, lyophilized SLA (containing 10 mg of proteins) was dissolved in 250 μl of buffer A (0.1% trifluoroacetic acetic acid, TFA) and the supernatant was then applied on an analytical reverse phase HPLC column, previously equilibrated with buffer A for 15 min. The elution of fractions was conducted using a linear gradient of buffer B (66.6% Acetonitrile in buffer A) and the chromatographic run was monitored at 280 nm of absorbance for 55 min. The fractions were collected based on their sharpness and their hydrophilic profile in the elution buffer. The samples were lyophilized and further solubilized with PBS, sterilized in a 0.22 μm membrane (Eppendorff, USA) and the protein amounts were estimated through the Bradford method. Lysis buffer was also applied onto an analytical reverse phase HPLC column as control sample under the same conditions of SLA. F1 was submitted to electrophoresis to determine the presence of proteins [27].

### Protein identification by mass spectrometry

Purified F1 was reduced, alkylated and submitted to in-gel digestion with trypsin [28]. An aliquot (4.5 μL) of the resulting peptide mixture was separated by C18 (100 μm × 100 mm) nanoUPLC (nanoAcquity, Waters) coupled with a Q-Tof Ultima mass spectrometer (Waters) with nano-electrospray source at a flow rate of 0.6 mL/min. The gradient was 2-90% acetonitrile in 0.1% formic acid over 45 min. The instrument was operated in the 'top three' mode, in which one MS spectrum was acquired followed by MS/MS of the top three most-intense peaks detected. The spectra were acquired using software MassLynx v.4.1 and the raw data files were converted to a peak list format (mgf) by the software Mascot Distiller v.2.3.2.0, 2009 (Matrix Science Ldt.) and searched against non-redundant protein database NCBI nr 2010.09.24 restricted to *Leishmania *(51635 sequences; 31433329 residues) using search engine MASCOT v.2.3.01 (Matrix Science Ltd.), with carbamidomethylation as a fixed modification, oxidation of methionine as a variable modification, one trypsin missed cleavage and a tolerance of 0.1 Da for both precursor and fragment ions.

### Immunization scheme and challenge

Male BALB/c mice, eight per group, were immunized with F1 fraction once a week for two consecutive weeks by subcutaneous route in the rump using 25 μg of protein [16,24]. Control mice (n = 16) were injected with 50 μl of PBS by the same route. One week after the last immunization, immunized mice were infected subcutaneously in the hind footpad with 10^6 ^promastigote forms of *L. (V.) shawi*. Eight mice from the control group received only PBS in the hind footpad and the remainder were infected using the same procedure for immunized mice. To investigate the long-term protection [29] induced by F1, mice were immunized as described above, and were challenged in the footpad after 4 months (F1-4 m group). The infection was monitored weekly for eight weeks by measuring the lesion size using a dial micrometer and expressed as the difference in size between the infected and the contra lateral uninfected footpad. The mice were sacrificed in a CO_2 _chamber and skin and lymph node fragments were obtained to determine parasite burden by the limiting-dilution assay [30]. Fragments of skin from the F1, F1-4 m, Infected and Healthy groups were processed by usual histological techniques to analyze the inflammatory process.

### Evaluation of cellular immune responses

Popliteal lymph nodes from F1, F1-4 m, Infected and Healthy groups were collected at 8 weeks postinfection and the cell suspensions (2 × 10^5^/well) were cultured under stimulation with 10 μg of AG or F1 fraction. After 72 h, the supernatants were collected and the amounts of IL-4, IL-12, IFN-γ, (BD, USA) and TGF-β (e-Biosciences, USA) were quantified by sandwich ELISA, in accordance with the manufacturer's recommendations, using recombinant cytokines as standard. Following supernatant collection, cell proliferation of each group was evaluated as described by Ahmed *et al. *(1994) [31]. Briefly, the plates with cells were washed 3 times with PBS and then 50 μl of 3-(4,5-Dimethylthiazol-2-yl)-2,5-diphenyltetrazolium bromide (MTT) at 5 mg/ml in RPMI 1640 was added to each well. After 4 h, 50 μl of 10% SDS was added to the wells. The plate was read in an ELISA reader at 595 nm.

### Analysis of memory T lymphocytes in immunized mice

Lymph node cells from F1, F1-4 m, Infected and Healthy groups were adjusted to 8 × 10^5 ^cell in a 96-well plates and monoclonal antibodies (BD, USA) anti-mouse CD3ε (FITC), anti-mouse CD4 L3T4 (PerCP), anti-mouse CD8α (Alexa Fluor 700), anti-mouse CD45RB (PE) and anti-mouse CD62L (APC) at a dilution of 1:100 were added to the cell culture and incubated for 30 min at 4°C. After this step, the cells were washed three times with MACS buffer (2 mM EDTA; 0.5% BSA em PBS) and the plate was centrifuged at 1700 rpm for 5 min at 4°C. The cells were fixed with 100 μl paraformaldehyde 1%, and the sample acquisitions were performed using a FACSCanto cytometer (BD, USA). To compensate samples, microbeads (BD, USA) stained with each monoclonal antibody was prepared and they were injected into the cytometer prior to the samples. The data were analyzed using FlowJo 7.6.1 (TreeStar, USA) for Windows. Both CD4^+ ^and CD8^+ ^T lymphocyte were identified in samples, followed by identification of CD62L^low ^and CD62L^high ^positive subsets. These T lymphocyte populations were further characterized by CD45RB marker [32] (Additional file 1: Figure S1).

### Analysis of the humoral immune response

The humoral immune response was evaluated by enzyme-linked immunosorbent assay (ELISA). High-binding plates (Costar, USA) were coated with 1 μg/well of *L. (V.) shawi *SLA, overnight at 4°C. Next, the plates were blocked with 10% of nonfat milk in PBS (2 h at 37°C) to prevent nonspecific binding. Mouse sera (1:50) were added and the plates were incubated for 1 h at 35°C. After washing, HPR goat anti-mouse IgG1 and IgG2a (SouthernBiotech, USA) (1:2000) were used for 1 h at 35°C. After washing, TMB was added to each well for 15 min. The reaction was stopped with 50 μl of 2 N sulfuric acid and the plates were read at 450 nm in an ELISA reader. Sera from *L. shawi *chronically infected mice were also used as positive controls (data not shown). PBS with 0.05% Tween 20 was used in all washing steps.

### Statistical analysis

The results were expressed as the mean ± standard deviation of three independent experiments and the nonparametric Mann-Whitney *U *test was used to compare lesion size, parasite load and cytokine expression between the groups. Differences were considered statistically significant at a 5% significance level (*p *< 0.05). Statistical analysis was performed using SPSS 17.0 for Windows software (SPSS Inc. USA).

## Results

### Purification of F1 fraction and brief summary of mass spectrometry data

**Figure 1:**
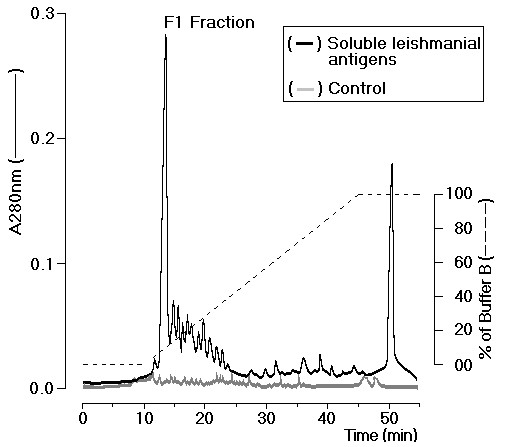
**Soluble leishmanial antigen (SLA) from *L. (V.) shawi *promastigotes was submitted to reverse phase chromatography and F1 proteic fraction were isolated based on their sharpness and hydrophilicity (black line)**. Lysis buffer also was applied as sample control (grey line).

The whole antigen was submitted to reverse phase HPLC to purify the fraction F1 (Figure 1, black line). The lysis buffer (control sample) did not interfere in the purification of F1 fraction (Figure 1, grey line). F1 fraction yield around one percent of SLA.

Due to the important data obtained using the F1 fraction, we considered it essential to identify the proteins present in this fraction. Mass spectrometry demonstrated that F1 presented 65 different proteins, 41 with acidic, 24 basic and 4 with a neutral pH. Thirty-two proteins belonging to F1 of *L. (V.) shawi *present similarities with proteins of *L. (V.) braziliensis*, 14 with proteins of *L. (L.) infantum*, 11 with proteins of *L. major*, 7 with proteins of *L. (L.) mexicana *and 1 with a protein of *L. donovani*. Other proteins had no previous reports in the literature concerning their functional activities and were considered hypothetical proteins (Additional file 2: Table S1).

### Lesion size, histology and analysis of parasite burden

**Figure 2:**
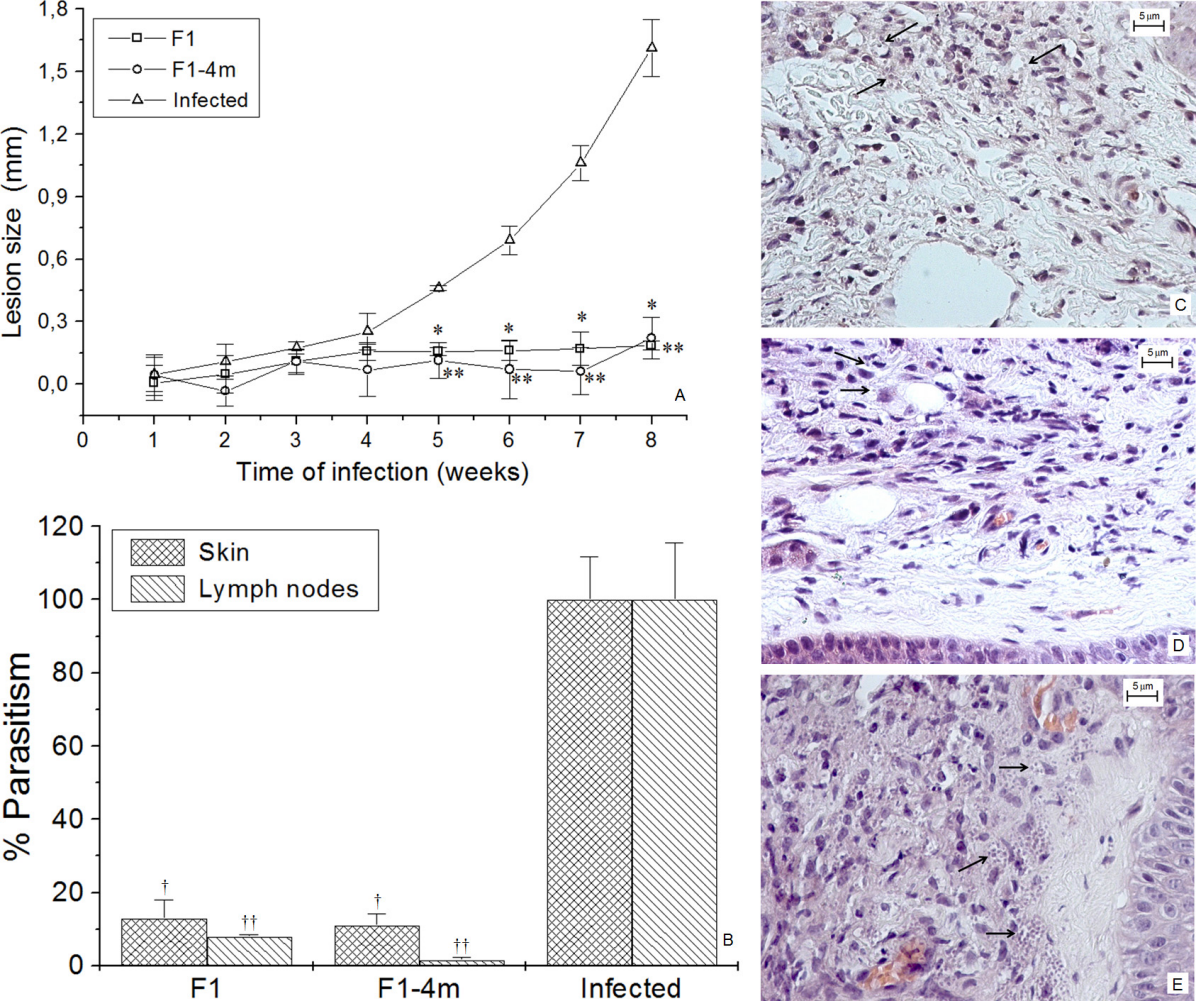
**Lesion development, parasitism and histopathology of F1, F1-4 m and Infected groups**. **A**--The course of lesions was accompanied during 8 weeks in all groups. **B**--Parasitism in skin and lymph nodes of F1, F1- 4 m and Infected groups. **C, D, E**--Histopathology of skin from F1, F1-4 m and Infected groups, respectively. Arrows show amastigote inner mononuclear cells. Coloration in HE at 400× magnification. Bars represent 5 μm. * and ** *p *< 0.05 indicate that lesion size of F1 and F1-4 m groups significantly differ from infected mice. ^†^and ^††^*p *< 0.05 indicate that parasite burden in the skin and lymph nodes of F1 and F1-4 m group significantly differ from infected mice.

The infected group presented a progressive increase in lesion size over time (Figure 2A), while the F1 and F1-4 m-immunized groups showed a decrease in skin lesions, which was significantly lower than infected mice between 5 and 8 weeks post-challenge (*p *< 0.05).

The F1-immunized group presented a reduction of 87% in parasites in the skin and 92% in the lymph nodes, while in F1-4 m-immunized mice, a reduction of 89.5% in the skin and 95.7% in the lymph nodes was recorded (Figure 2B).

The skin of the F1 and F1-4 m groups presented focal mononuclear inflammatory infiltrate with no necrotic region and macrophages with few parasites (Figure 2C and 2D, arrows). In contrast, the infected group presented diffuse inflammatory infiltrate into the epidermis and dermis, with heavily parasitized mononuclear cells (Figure 2E).

### Analysis of cellular immune response

**Figure 3:**
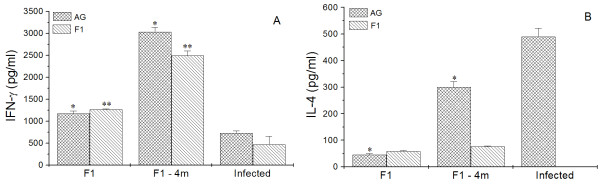
**The levels of IFN-γ (A) and IL-4 (B) were quantified in supernatant of lymph node cells from F1, F1-4 m and Infected groups**. * and **p < 0.05 indicate that cytokine production from cells stimulated with AG or F1 significantly differs from cytokine production from the infected group under the same stimulations.

Lymph node cells from the F1 and F1-4 m groups under stimulation with either AG or F1 fraction produced greater levels of IFN-γ compared with cells from infected mice (*p *< 0.05) stimulated with the same antigens (Figure 3A).

In comparison with the infected group, AG-stimulated cells from the F1 and F1-4 m groups presented diminished production of IL-4 (*p *< 0.05); however, the stimulation of cells from the F1 and F1-4 m groups with F1 fraction led to the increase of this cytokine (*p *< 0.05), which was not detected in cells from infected mice under F1 fraction stimulation (Figure 3B).

Cells from healthy mice produced no significant amounts of cytokines. Similarly, cells from all groups with no specific stimulation produced no IL-4 and IFN-γ. IL-12 and TGF-β proteins were not detected with or without specific stimulations.

**Figure 4:**
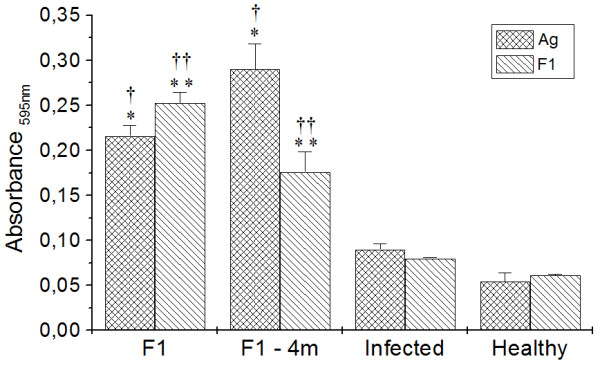
**Cell proliferation was assayed in F1, F1-4 m, Infected and Healthy groups**. * and ** *p *< 0.05 indicate that cellular proliferation of cells stimulated with AG and F1 significantly differ from cells from the infected group under the same stimulations. ^† ^and ^†† ^*p *< 0.05 indicates that cellular proliferation of cells stimulated with AG and F1 significantly differ from the Healthy group under the same stimulations.

After stimulation with AG and F1 fraction, cell proliferations were assayed using MTT (Figure 4). The F1 and F1-4 m groups presented significantly greater proliferation under any stimuli compared with cells from both infected and healthy mice (*p *< 0.05).

### Analysis of memory T lymphocytes in immunized mice

**Figure 5:**
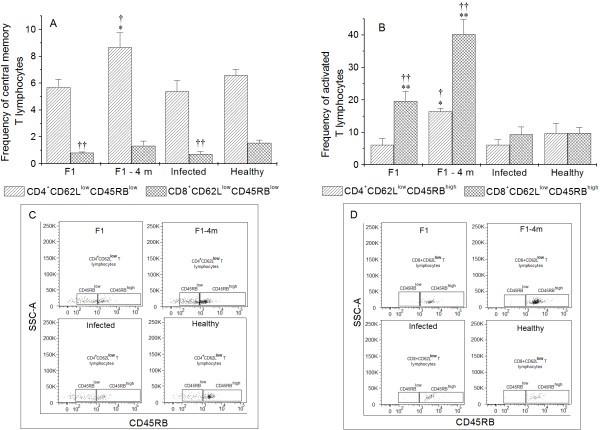
**Analysis of memory cells in F1, F1-4 m, Infected and Healthy groups**. *p < 0.05 indicates statistically significant differences between the frequencies of CD4^+^CD62L^low^CD45RB^low ^and CD4^+^CD62L^low^CD45RB^high ^T lymphocytes compared with the frequencies of the infected group. **p < 0.05 indicates statistically significant differences between the frequencies of CD8^+^CD62L^low^CD45RB^high ^T lymphocytes compared with the frequencies of the infected group. ^†^*p *< 0.05 indicates that the frequencies of CD4^+^CD62L^low^CD45RB^low ^and CD4^+^CD62L^low^CD45RB^high ^T lymphocytes significantly differ compared with the frequencies of the healthy group. ^††^*p *< 0.05 indicates that the frequencies of CD8^+^CD62L^low^CD45RB^low ^and CD8^+^CD62L^low^CD45RB^high ^T lymphocytes significantly differ compared with the frequencies of the infected group.

The F1-4 m group presented a significant increase (*p *< 0.05) in CD4^+^CD62L^low^CD45RB^low ^T lymphocytes (CD4^+^T central memory lymphocytes) compared with infected and healthy groups (Figure 5A). Both the F1 and infected groups presented significant reductions in the frequencies of CD8^+ ^T memory central cells (CD8^+^CD62L^low^CD45RB^low^).

F1-4 m presented a significant increase in CD3^+^CD4^+^CD62L^low^CD45RB^high ^(activated CD4^+ ^T lymphocytes) (Figure 5B) compared with infected and healthy mice (*p *< 0.05), and both F1 and F1-4 m presented significant increases in activated CD8+ T cells (CD3^+^CD8^+^CD62L^low^CD45RB^high^) compared with infected and healthy mice.

The frequencies of naïve T CD4^+ ^and CD8^+ ^lymphocytes (CD3^+^CD4^+^CD62L^high ^or CD3^+^CD8^+^CD62L^high^) were similar between the groups (data not show).

### Analysis of humoral immune response

**Figure 6:**
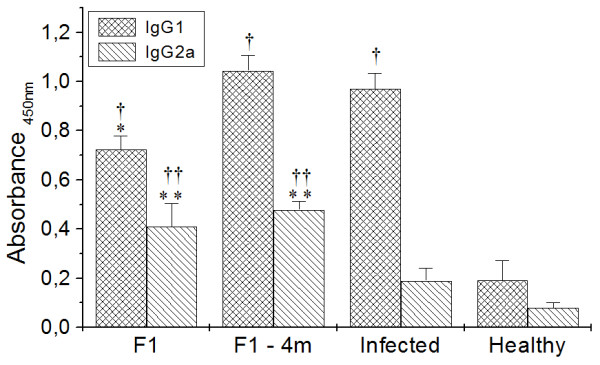
**Humoral immune response in F1, F1-4 m, Infected and Healthy mice**. * and **p < 0.05 indicate that IgG1 and IgG2a anti-*Leishmania *significantly differ from the infected group, ^† ^and ^††^*p *< 0.05 indicate that IgG1 and IgG2a anti-*Leishmania *significantly differ from the healthy group.

All the experimental groups presented increases in IgG1 anti-*Leishmania *antibodies compared with healthy mice (*p *< 0.05); however, the F1-immunized group presented a significant reduction in IgG1 anti-*Leishmania *compared with infected mice (Figure 6). The levels of IgG2a anti-*Leishmania *were increased in both the F1 and F1-4 m groups compared with infected and healthy mice (*p *< 0.05).

## Discussion

In the present study the antigenic fraction (F1) derived from *L. (V.) shawi *promastigotes was purified, characterized and its protective potential in a murine model of *L. (V.) shawi *infection was evaluated. The F1 fraction obtained seems to provide long-term protection for mice.

Characterization of the F1 fraction revealed its complexity. The fraction was composed of a variety of proteins that had no previously reported function, but these can be associated with physiological requirements of *L. (V.) shawi*. Certain proteins seem to participate in the physiology and the infective processes of this parasite, such as α-tubulin, elongation factor 1-α and glucose transporter (details in Additional file 2: Table S1) and a few of them had some immunostimulatory properties, such as dynein [33], chaperonin HSP60 [34] and kinesin [35]. Dey *et al. *[36] verified that a DNA vaccine composed of kinesin and esat-6 genes from *L. donovani *and *Mycobacterium tuberculosis*, respectively, presented high immunogenicity to BALB/c mice as determined by its potential to induce IL-2 and IFN-γ production, indicating that kinesin protein presented in F1 could participate in the immunity generation of F1 and F1-4 m-immunized mice; however, some proteins could be associated with the suppression of cellular immune response due to the diversity of F1. In addition, the F1 fraction showed a high number of proteins similar to those of *L. (V.) braziliensis*. Antigens with previously reported immunostimulatory properties showed a high degree of genetic similarities with those of *L. (V.) braziliensis *(98-100%). On the other hand, antigens from *L. (V.) shawi *presented some degree of genetic heterogenicity (68-89%) compared with antigens of *Leishmania *subgenus representatives (data not shown). These findings suggest that certain antigens of the F1 fraction could be interesting targets for the development of a cross-protective vaccine against infections caused by the *Viannia *subgenus due to the high degree of similarity. However, this proposal needs to be confirmed in future experiments.

In experimental *L. (V.) shawi *infection, progressive disease is associated with greater lesion size, strong inflammatory processes with necrotic areas and heavily parasitized mononuclear cells [22,30]. In the F1 fraction, certain proteins may be protective, since F1-immunized mice were able to control the infection, as determined by the smaller lesion size, mild inflammatory processes and, importantly, they drastically reduced the number of parasites in the skin and lymph nodes of mice. These characteristics were reproduced in mice that were challenged 4 months after the last immunization, suggesting that as well as immunogenicity and protection, the F1 fraction induced long-term protection.

In fact, lymph node cells from the groups immunized with the F1 fraction increased the levels of IFN-γ and reduced production of IL-4 following AG stimulation. To eliminate parasites, it is essential to increase IFN-γ levels, since this cytokine is capable of activating macrophages to a leishmanicidal state through the production of microbicidal compounds, such as nitric oxide, and upregulate the expression of MHC [37], permitting elimination of the infectious agent and amplification of the expression of antigens to lymphocytes. Concomitantly, a low level of IL-4 is highly important to achieve resistance, since it can be viewed as a suppressor and mediator of Th1 and Th2 immune responses, respectively [38]. In addition, some studies of vaccination using second-generation vaccine candidates demonstrated that protection was associated with an increase in IFN-γ and a decrease in IL-4 cytokines in mice [39], indicating that immunogens capable of stimulating this immunological profile could be important vaccine candidates.

The stimulation of lymph node cells from F1- and F1-4 m-immunized mice with F1 fraction led to an increase in IFN-γ and IL-4 levels. Although IL-4 has been accepted as inductor of susceptibility in leishmaniasis, some studies have shown its importance in the generation, proliferation and maintenance of memory cells in experimental infections by *L. donovani *and *Plasmodium yoelii *[40,41]. In fact, cells from F1 and F1-4 m showed increased proliferation compared with cells from infected mice, indicating that IL-4 could be partly responsible for this activity. In addition, in nonimmunized mice, cell proliferation did not occur, suggesting immune suppression caused by high parasitism, like that observed for *L. donovani *and *L. major *species [42,43]. Besides the role of IFN-γ in the activation of infected cells, it also could play an important role in T lymphocyte proliferation [44].

After their encounter with antigens, lymphocytes are activated and proliferate, and after their clearance by regulatory mechanisms, the quantity of lymphocytes decreases, resulting in a small fraction of surviving memory cells that readily proliferate following a new encounter with the antigen [45]. In order to evaluate the frequencies of memory T cells in immunized mice, T lymphocytes were phenotyped with the markers CD62L and CD45RB. CD62L is an adhesion molecule associated with the activation and circulation of activated lymphocytes [46,47]. CD45RB is an important molecule associated with TCR activation [48] and its expression is reduced following T lymphocyte activation. For these reasons, it is possible to assume that T lymphocytes presenting the phenotype CD62L^low^CD45RB^low ^are central memory cells and CD62L^low^CD45RB^high ^are activated T cells [32,49]. In this study, the F1 group presented a low frequency of central memory CD8^+ ^T cells and a high frequency of activated CD8^+ ^T lymphocytes, a reduction that could be associated with the differentiation of CD8^+^CD62L^low^CD45RB^low ^toward an activated phenotype. Similar results were viewed in the F1-4 m group; however, without the decrease in the frequency of central memory CD8^+ ^T cells. This activated phenotype of CD8^+^T lymphocytes should allow traffic between homing lymphoid organs to the site of infection, where this cell population can eliminate infected cells, since the main characteristic of CD8^+^T lymphocyte is to eliminate the target cell by cytotoxicity [50,51]. Moreover, vaccination studies have stated that CD8^+^T cells activated by immunogens are primordial in the resistance against intracellular infections, establishing long-term immunity [29,52]. Thus, immunization with the F1 fraction should activate CD8^+^T cells, leading to resistance against *L. (V.) shawi *infection.

The F1-4 m group also showed an increase in both central and activated CD4^+ ^T lymphocytes, which was not observed in the F1 group. This fact can be associated with a high frequency of CD8^+ ^T lymphocytes in F1-4 m, since activation of high numbers of CD8^+^T cells can also activate CD4^+^T lymphocytes, increasing the production of IFN-γ [53], which was observed in stimulated cells of F1-4 m mice.

The increase in IL-4 and IFN-γ can have direct consequences in elevating IgG1 and IgG2a, respectively [54]. These isotypes seem to have a direct correlation between susceptibility and resistance in experimental leishmaniasis, since IgG1 is linked to humoral immune response, while IgG2a is associated with the cellular immune response [55] and parasite destruction, which taken together, support the present results in which F1-immunized mice tend towards resistance induced by immunization with the F1 fraction, while the infected group tends towards susceptibility.

## Conclusions

Analysis of our results indicates that the F1 fraction induces a beneficial response in BALB/c mice. The antigenic fraction favored increased production of IFN-γ, which has been associated with resistance against infection. Moreover, the long-term protection observed in immunized mice could be associated with both central memory and activated CD4^+ ^and CD8^+ ^T lymphocytes. The results presented herein suggest that resistance against *L. (V.) shawi *infection can occur through activation of both CD4^+ ^and CD8^+ ^T lymphocytes. Antigens capable of inducing this immunological profile could be important targets in the development of vaccine candidates against American Tegumentary Leishmaniasis.

## Competing interests

The authors declare that they have no competing interests.

## Authors' contributions

All the authors contributed equally to the present study and also read and approved the final manuscript.

## Supplementary Material

Additional file 1 **Figure S1**. Flow cytometry strategy used to analyze cell populations. CD4+ and CD8 + T lymphocytes were identified, followed by their characterization of CD62L low and high populations. The levels of expression of CD45RB were analyzed in the population with high and low expression of the markers CD62L. Click here for file

Additional file 2 **Table S1**. Proteins detected in F1 antigen purified through reverse phase HPLC. Click here for file
