# Supplementary material for: Proteins of Leishmania (Viannia) shawi confer protection associated with Th1 immune response and memory generation
Source: Parasit Vectors. 2012 Mar 30;5:64. doi: 10.1186/1756-3305-5-64 (PMC3342111; doi:10.1186/1756-3305-5-64)
Supplement: Additional file 1 — Figure S1. Flow cytometry strategy used to analyze cell populations. CD4+ and CD8 + T lymphocytes were identified, followed by their characterization of CD62L low and high populations. The levels of expression of CD45RB were analyzed in the population with high and low expression of the markers CD62L. [file 1756-3305-5-64-S1.DOCX]

Table I – Proteins detected in F1 antigen purified through reverse phase HPLC

| Protein identification (NCBI reference sequence) | Parasite | Function^a^ | kDa/pI | Reference |
| --- | --- | --- | --- | --- |
| Short chain dehydrogenase ([XP_001568225.1](http://www.ncbi.nlm.nih.gov/protein/154344567?report=genbank&log$=prottop&blast_rank=1&RID=KYCWUATU01N)) | *L. braziliensis* | 1, 4 | 28.1/ 7.6 | [36] |
| Ribosomal protein s20 ([XP_001566139.1](http://www.ncbi.nlm.nih.gov/protein/154340365?report=genbank&log$=prottop&blast_rank=1&RID=KYD1REDK01S)) | *L. braziliensis* |  | 13.1/ 9.6 |  |
| Alpha tubulin ([XP_001566612.1](http://www.ncbi.nlm.nih.gov/protein/154341318?report=genbank&log$=prottop&blast_rank=1&RID=KYE5TNWH01N)) | *L. braziliensis* | 2 | 28.8/ 5.0 | [37] |
| Hypothetical protein([XP_001566360.1](http://www.ncbi.nlm.nih.gov/protein/154340814?report=genbank&log$=prottop&blast_rank=1&RID=KY8N689501S)) | *L. braziliensis* |  | 318.1/5.5 |  |
| Beta galactofuranosyl glycosyle transferase ([XP_001564335.1](http://www.ncbi.nlm.nih.gov/protein/154336199?report=genbank&log$=prottop&blast_rank=1&RID=KY90SZ8B01S)) | *L. braziliensis* |  | 56.2/ 6.7 |  |
| Hypothetical protein ([XP_001566489.1](http://www.ncbi.nlm.nih.gov/protein/154341072?report=genbank&log$=prottop&blast_rank=1&RID=KY93D0FH011)) | *L. braziliensis* |  | 69.0/ 5.0 |  |
| Hypothetical protein ([XP_001566489.1](http://www.ncbi.nlm.nih.gov/protein/154341072?report=genbank&log$=prottop&blast_rank=1&RID=U759BDR9015)) | *L. braziliensis* |  | 69.0/ 5.0 |  |
| Hypothetical protein (X[P_001564809.1](http://www.ncbi.nlm.nih.gov/protein/154337152?report=genbank&log$=prottop&blast_rank=1&RID=KY98TCA501S)) | *L. braziliensis* |  | 75.8/ 8.8 |  |
| Dead/h helicase ([XP_001568924.1](http://www.ncbi.nlm.nih.gov/protein/154345974?report=genbank&log$=prottop&blast_rank=1&RID=KY99MSFB01N)) | *L. braziliensis* |  | 122.7/5.4 |  |
| Phospholipid transporting ATPase-like protein  ([XP_001562679.1](http://www.ncbi.nlm.nih.gov/protein/154332834?report=genbank&log$=prottop&blast_rank=1&RID=KY9CA3SK01N)) | *L. braziliensis* |  | 264.4/7.6 |  |
| Hypotetical protein ([XP_001563405.1](http://www.ncbi.nlm.nih.gov/protein/154334307?report=genbank&log$=prottop&blast_rank=1&RID=KY9CSW0H01S)) | *L. braziliensis* |  | 16.7/ 9.4 |  |
| Vacuolar ATPase subunit-like protein ([XP_001561928.1](http://www.ncbi.nlm.nih.gov/protein/154332223?report=genbank&log$=prottop&blast_rank=1&RID=KY9F5SN601S)) | *L. braziliensis* | 2 | 41.6/ 5.0 | [37] |
| Zinc-finger protein ZPR1([XP_001563921.1](http://www.ncbi.nlm.nih.gov/protein/154335362?report=genbank&log$=prottop&blast_rank=1&RID=KY9FNU9V01N)) | *L. braziliensis* | 2 | 54.3/ 4.5 | [38] |
| Elongation factor 1-alpha ([XP_001563777.1](http://www.ncbi.nlm.nih.gov/protein/154335074?report=genbank&log$=prottop&blast_rank=1&RID=KY9JWRWA01N)) | *L. braziliensis* | 2 | 49.1/9.0 | [39] |
| Unnamed protein product ([CBZ31415.1](http://www.ncbi.nlm.nih.gov/protein/322496344?report=genbank&log$=prottop&blast_rank=1&RID=U75Z10UX014)) | *L. donovani* |  | 18.2/4.4 |  |
| Hypothetical protein ([XP_001467027.1](http://www.ncbi.nlm.nih.gov/protein/146093832?report=genbank&log$=prottop&blast_rank=2&RID=U762B606014)) | *L. infantum* |  | 590.0/6.3 |  |
| Hypothetical protein ([XP_001562346.1](http://www.ncbi.nlm.nih.gov/protein/154339309?report=genbank&log$=prottop&blast_rank=1&RID=KY9R7WAW01N)) | *L. braziliensis* |  | 186.5/6.7 |  |
| Hypothetical protein ([XP_001683088.1](http://www.ncbi.nlm.nih.gov/protein/157869072?report=genbank&log$=prottop&blast_rank=1&RID=U76HAXYP014)) | *L. major* |  | 188.5/8.9 |  |
| Hypothetical protein ([XP_001569257.1](http://www.ncbi.nlm.nih.gov/protein/154346640?report=genbank&log$=prottop&blast_rank=1&RID=KY9YEXPR016)) | *L. braziliensis* |  | 71.3/9.6 |  |
| Dynein ([XP_001567473.1](http://www.ncbi.nlm.nih.gov/protein/154343055?report=genbank&log$=prottop&blast_rank=1&RID=KY9ZJASX014)) | *L. braziliensis* | 2, 5 | 68.0/5.5 | [40; 41] |
| Inositol polyphosphate kinase-like protein ([XP_001687633.1](http://www.ncbi.nlm.nih.gov/protein/157866483?report=genbank&log$=prottop&blast_rank=1&RID=U76MTC8T01N)) | *L. major* |  | 116.0/6.3 |  |
| Hypothetical protein ([CBZ25880.1](http://www.ncbi.nlm.nih.gov/protein/322490618?report=genbank&log$=prottop&blast_rank=2&RID=U76R055X01S)) | *L. mexicana* |  | 47.9/5.8 |  |
| Phosphonopyruvate decarboxylase-like protein  ([[CBZ28529.1](http://www.ncbi.nlm.nih.gov/protein/322493244?report=genbank&log$=prottop&blast_rank=2&RID=U76RD4HN01S)](http://www.ncbi.nlm.nih.gov/protein/154340497?report=genbank&log$=prottop&blast_rank=1&RID=KYA54A7W011) ) | *L. mexicana* |  | 44.4/5.7 |  |
| Hypothetical protein ([XP_001684321.1](http://www.ncbi.nlm.nih.gov/protein/157871544?report=genbank&log$=prottop&blast_rank=1&RID=U76TPBEY015)) | *L. major* |  | 83.0/6.5 |  |
| Hypothetical protein ([XP_001566616.1](http://www.ncbi.nlm.nih.gov/protein/154341326?report=genbank&log$=prottop&blast_rank=3&RID=U76U5APJ015)) | *L. braziliensis* |  | 119.9/6.1 |  |
| Hypothetical protein ([XP_001682227.1](http://www.ncbi.nlm.nih.gov/protein/157867345?report=genbank&log$=prottop&blast_rank=1&RID=U76XSVYP01S)) | *L. major* |  | 157.0/8.4 |  |
| AAA family ATPase-like protein ([CBZ28930.1](http://www.ncbi.nlm.nih.gov/protein/322493640?report=genbank&log$=prottop&blast_rank=1&RID=U76Y8B8001S)) | *L. mexicana* |  | 83.3/5.9 |  |
| Hypothetical protein ([XP_001470535.1](http://www.ncbi.nlm.nih.gov/protein/146090030?report=genbank&log$=prottop&blast_rank=2&RID=U773212101S) ) | *L. infantum* |  | 89.0/4.9 |  |
| L-gulonolactone oxidase ([XP_001464804.1](http://www.ncbi.nlm.nih.gov/protein/146083657?report=genbank&log$=prottop&blast_rank=3&RID=U773FZDN01S)) | *L. infantum* |  | 55.4/6.4 |  |
| Putative chaperonin HSP60 ([[CAM42998.2](http://www.ncbi.nlm.nih.gov/protein/322505537?report=genbank&log$=prottop&blast_rank=1&RID=U778D5WR014)](http://www.ncbi.nlm.nih.gov/protein/154343225?report=genbank&log$=prottop&blast_rank=1&RID=KYAF7V2K016) ) | *L. braziliensis* | 2, 5 | 64.3/6.3 | [42; 43] |
| ATP-dependent DEAD/H DNA helicase recQ ([[XP_001465982.1](http://www.ncbi.nlm.nih.gov/protein/146088064?report=genbank&log$=prottop&blast_rank=2&RID=U778S9R4015)](http://www.ncbi.nlm.nih.gov/protein/154338347?report=genbank&log$=prottop&blast_rank=1&RID=KYAJ54TW011) ) | *L. infantum* | 1 | 204.4/6.9 | [37] |
| Hypothetical protein ([XP_001567022.1](http://www.ncbi.nlm.nih.gov/protein/154342148?report=genbank&log$=prottop&blast_rank=1&RID=KYAJNYZV014)) | *L. braziliensis* |  | 68.9/5.7 |  |
| Hypothetical protein ([XP_001566994.1](http://www.ncbi.nlm.nih.gov/protein/154342085?report=genbank&log$=prottop&blast_rank=1&RID=KYANKMBJ014)) | *L. braziliensis* |  | 50.6/6.0 |  |
| Kinesin ([XP_001467156.1](http://www.ncbi.nlm.nih.gov/protein/146094090?report=genbank&log$=prottop&blast_rank=4&RID=U77U1RR6014) ) | *L. infantum* | 2, 5 | 133.7/5.3 | [44] |
| ABC transporter ([XP_001463924.1](http://www.ncbi.nlm.nih.gov/protein/146079998?report=genbank&log$=prottop&blast_rank=3&RID=U77UFSM101S)) | *L. infantum* | 1, 4 | 199.7/5.9 | [45; 46] |
| Hypothetical protein ([XP_001563144.1](http://www.ncbi.nlm.nih.gov/protein/154333775?report=genbank&log$=prottop&blast_rank=1&RID=KYATGTMP01S)) | *L. braziliensis* |  | 82.2/6.8 |  |
| Hypothetical protein ([XP_001565070.1](http://www.ncbi.nlm.nih.gov/protein/154337675?report=genbank&log$=prottop&blast_rank=1&RID=KYAXC4W601N)) | *L. infantum* |  | 302.7/5.9 |  |
| Hypothetical protein ([XP_001680850.1](http://www.ncbi.nlm.nih.gov/protein/157864279?report=genbank&log$=prottop&blast_rank=1&RID=U7824VWX014)) | *L. major* |  | 110.6/10.2 |  |
| Hypothetical protein ([XP_001569024.1](http://www.ncbi.nlm.nih.gov/protein/154346174?report=genbank&log$=prottop&blast_rank=1&RID=KYB2G8KD01N)) | *L. braziliensis* |  | 14.1/6.7 |  |
| Hypothetical protein ([XP_001687333.1](http://www.ncbi.nlm.nih.gov/protein/157879007?report=genbank&log$=prottop&blast_rank=1&RID=U7883XAZ01S)) | *L. major* |  | 51.1/8.9 |  |
| Conserved hypothetical protein ([CBZ24366.1](http://www.ncbi.nlm.nih.gov/protein/322489114?report=genbank&log$=prottop&blast_rank=2&RID=U78ENG1X01S)) | *L. mexicana* |  | 707.2/5.2 |  |
| Hypothetical protein ([CAB71235.1](http://www.ncbi.nlm.nih.gov/protein/6855412?report=genbank&log$=prottop&blast_rank=2&RID=U78F3XPM01S)) | *L. major* |  | 116.3/6.3 |  |
| Telomerase reverse transcriptase ([XP_001469622.1](http://www.ncbi.nlm.nih.gov/protein/146103683?report=genbank&log$=prottop&blast_rank=2&RID=U78J4B4D015)) | *L. infantum* |  | 157.5/10.4 |  |
| Hypothetical protein ([XP_001563929.1](http://www.ncbi.nlm.nih.gov/protein/154335381?report=genbank&log$=prottop&blast_rank=1&RID=KYBD12H5011)) | *L. braziliensis* |  | 113.1/6.3 |  |
| Protein phosphatase 2C-like protein ([XP_001563376.1](http://www.ncbi.nlm.nih.gov/protein/154334249?report=genbank&log$=prottop&blast_rank=1&RID=KYBCW32K01S)) | *L. braziliensis* |  | 99.6/9.4 |  |
| Conserved hypothetical protein ([CBZ28153.1](http://www.ncbi.nlm.nih.gov/protein/322492874?report=genbank&log$=prottop&blast_rank=1&RID=U78XXDD501S)) | *L. mexicana* |  | 74.8/6.5 |  |
| Hypothetical protein ([XP_001463831.1](http://www.ncbi.nlm.nih.gov/protein/146079686?report=genbank&log$=prottop&blast_rank=2&RID=U791AX8V015)) | *L. infantum* |  | 498.3/7.3 |  |
| Conserved hypothetical protein ([CBZ29607.1](http://www.ncbi.nlm.nih.gov/protein/322494308?report=genbank&log$=prottop&blast_rank=1&RID=U791NB5H014)) | *L. mexicana* |  | 43.8/9.4 |  |
| Hypothetical protein ([XP_001466453.1](http://www.ncbi.nlm.nih.gov/protein/146091131?report=genbank&log$=prottop&blast_rank=3&RID=U79400XN015)) | *L. infantum* |  | 54.8/5.8 |  |
| Hypothetical protein ([CAM67008.2](http://www.ncbi.nlm.nih.gov/protein/321399260?report=genbank&log$=prottop&blast_rank=2&RID=U794HT14015)) | *L. infantum* |  | 230.7/8.7 |  |
| Hypothetical protein ([XP_001562498.1](http://www.ncbi.nlm.nih.gov/protein/154332248?report=genbank&log$=prottop&blast_rank=1&RID=KYBY8D8Z011)) | *L. braziliensis* |  | 33.6/6.9 |  |
| Glucose transporter, lmgt1 ([CBZ26490.1](http://www.ncbi.nlm.nih.gov/protein/322491224?report=genbank&log$=prottop&blast_rank=1&RID=U798SR5N015)) | *L. mexicana* | 1, 3 | 66.1/7.7 | [47; 48] |
| Protein transport protein sec31 ([XP_001681582.1](http://www.ncbi.nlm.nih.gov/protein/157865750?report=genbank&log$=prottop&blast_rank=1&RID=U79BX85X015)) | *L. major* |  | 122.1/6.1 |  |
| Hypothetical protein ([XP_001562957.1](http://www.ncbi.nlm.nih.gov/protein/154333400?report=genbank&log$=prottop&blast_rank=1&RID=KYBZW5F0011)) | *L. braziliensis* |  | 74.2/ 8.9 |  |
| Conserved hypothetical protein ([CAM68929.2](http://www.ncbi.nlm.nih.gov/protein/321398396?report=genbank&log$=prottop&blast_rank=2&RID=U79EWVB2015)) | *L. infantum* |  | 262.1/ 9.8 |  |
| Hypothetical protein ([XP_001683017.1](http://www.ncbi.nlm.nih.gov/protein/157868930?report=genbank&log$=prottop&blast_rank=1&RID=U79FAB0W01N)) | *L. infantum* |  | 149.7/ 5.4 |  |
| Hypothetical protein ([XP_001568247.1](http://www.ncbi.nlm.nih.gov/protein/154344611?report=genbank&log$=prottop&blast_rank=1&RID=KYC5NS9K01S)) | *L. braziliensis* |  | 341.0/ 5.0 |  |
| Myo-inositol-1(or 4)-monophosphatase 1([XP_001563919.1](http://www.ncbi.nlm.nih.gov/protein/154335358?report=genbank&log$=prottop&blast_rank=1&RID=KYC6051401N)) | *L. braziliensis* | 2 | 29.6/ 5.2 | [37] |
| Hypothetical protein ([X [XP_001683215.1](http://www.ncbi.nlm.nih.gov/protein/157869327?report=genbank&log$=prottop&blast_rank=1&RID=U79N2M4M01N)](http://www.ncbi.nlm.nih.gov/protein/154337487?report=genbank&log$=prottop&blast_rank=1&RID=KYCBBNHC011) ) | *L. major* |  | 185.6/6.7 |  |
| Hypothetical protein ([XP_001561744.1](http://www.ncbi.nlm.nih.gov/protein/154331854?report=genbank&log$=prottop&blast_rank=1&RID=KYCD934U014)) | *L. braziliensis* |  | 171.4/ 6.1 |  |
| Hypothetical protein ([XP_001563774.1](http://www.ncbi.nlm.nih.gov/protein/154335055?report=genbank&log$=prottop&blast_rank=1&RID=KYCJ19T801S)) | *L. braziliensis* |  | 176.5/ 9.2 |  |
| Hypothetical protein ([XP_001463402.1](http://www.ncbi.nlm.nih.gov/protein/146077974?report=genbank&log$=prottop&blast_rank=2&RID=U79VBUA501S)) | *L. infantum* |  | 234.3/ 6.8 |  |
| Hypothetical protein ([XP_001685684.1](http://www.ncbi.nlm.nih.gov/protein/157874401?report=genbank&log$=prottop&blast_rank=1&RID=U79VS9T701S)) | *L. major* |  | 130.8/ 5.9 |  |
| Hypothetical protein ([XP_001683585.1](http://www.ncbi.nlm.nih.gov/protein/157870069?report=genbank&log$=prottop&blast_rank=1&RID=U79ZCPYZ01N)) | *L. major* |  | 105.4/ 8.8 |  |
| Conserved hypothetical protein ([CAM42931.2](http://www.ncbi.nlm.nih.gov/protein/322505509?report=genbank&log$=prottop&blast_rank=1&RID=U79ZN2NA015)) | *L. braziliensis* |  | 90.3/ 5.6 |  |

^a^ Function of antigens. 1 – Metabolism; 2 – Physiology; 3 – Virulence; 4 – Drug resistance; 5 – Immunostimulatory.
