# Supplementary figures and images for: Proteins of Leishmania (Viannia) shawi confer protection associated with Th1 immune response and memory generation
Source: Parasit Vectors. 2012 Mar 30;5:64. doi: 10.1186/1756-3305-5-64 (PMC3342111; doi:10.1186/1756-3305-5-64)

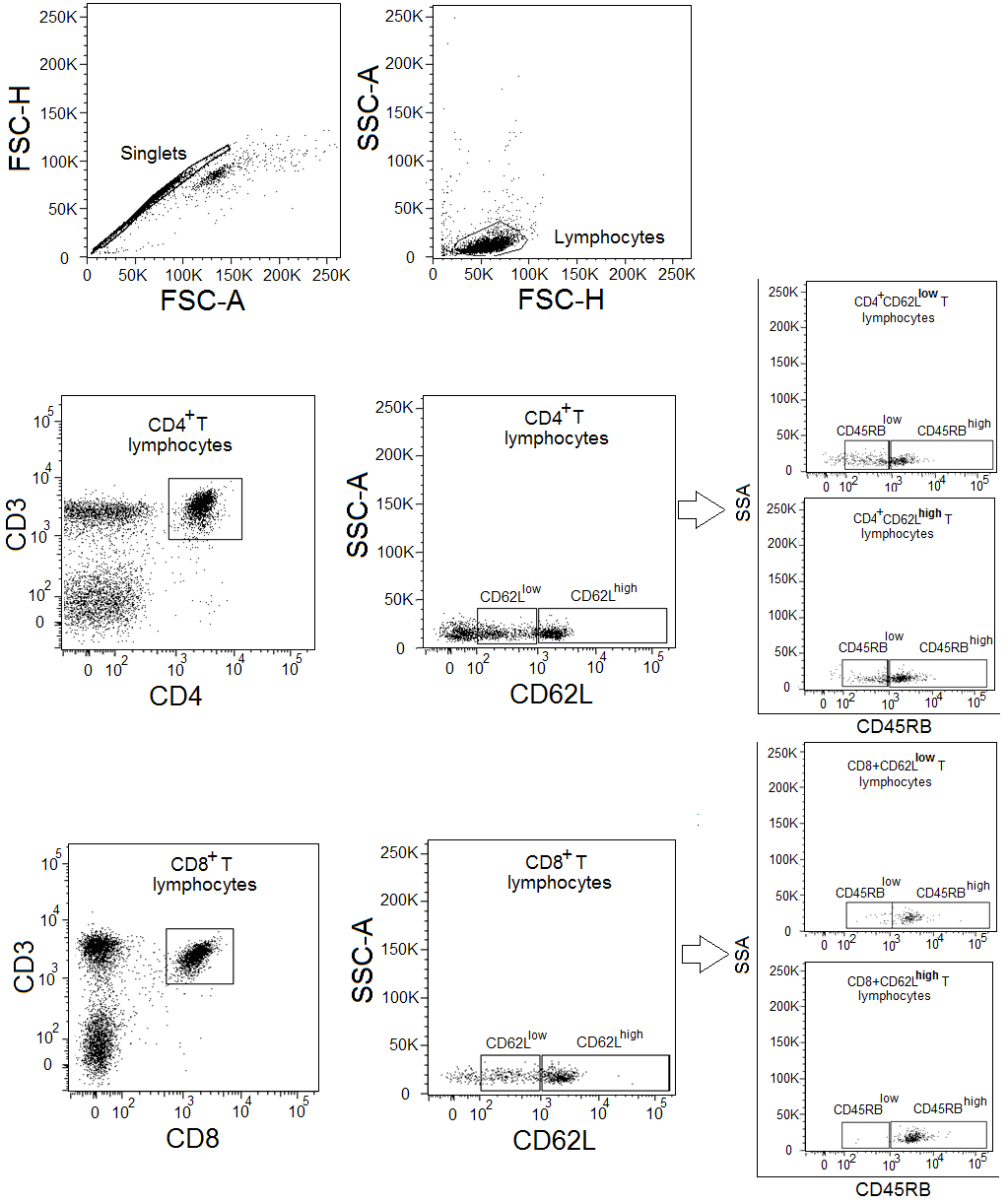

Supplement: Additional file 2 — Table S1. Proteins detected in F1 antigen purified through reverse phase HPLC. [file 1756-3305-5-64-S2.TIFF]
